# Internet-based cognitive behavioral therapy for prevention of depression during pregnancy and in the postpartum period

**DOI:** 10.1192/j.eurpsy.2023.394

**Published:** 2023-07-19

**Authors:** D. Nishi, K. Imamura, K. Watanabe, E. Obikane, N. Sasaki, N. Yasuma, Y. Sekiya, Y. Matsuyama, N. Kawakami

**Affiliations:** ^1^The University of Tokyo, Tokyo; ^2^Kitasato University, Sagamihara; ^3^National Center for Child Health and Development, Tokyo; ^4^Ageonomori Clinic, Ageo, Japan

## Abstract

**Introduction:**

Prevention of perinatal depression beginning from the antenatal period is essential.

**Objectives:**

This study aimed to investigate the effectiveness of recently developed internet-delivered cognitive behavioral therapy (iCBT) for preventing the onset of a major depressive episode (MDE) in the third trimester and at 3 months postpartum.

**Methods:**

This is a two-arm, parallel-group, general-information controlled, randomized controlled trial. Participants were 5,017 pregnant women at 16–20 weeks’ gestation without MDE at baseline. They were randomly assigned to an iCBT (intervention; n = 2,509) or general-information (control; n = 2,508) group, stratified by psychological distress at baseline. The primary outcomes were the numbers of new MDE onsets, measured using the World Health Organization Composite International Diagnostic Interview 3.0, at 32 weeks’ gestation and at 3 months postpartum.

**Results:**

New MDE onset was reported by 59 participants (2.35%) in the intervention group and 73 (2.91%) in the control group during follow-up. Compared with the control group, the hazard ratio (HR) of MDE in the intervention group was 0.85 (95% CI 0.61–1.20). Among participants who scored between 5 and 8 on K6 at baseline, 10 (1.37%) in the intervention group reported new onset of MDE, compared with 28 (3.81%) in the control group, and the HR of MDE was 0.38 (95%CI 0.19–0.79).

**Conclusions:**

No intervention effect was found for iCBT in preventing new onset of perinatal MDE. iCBT might prevent perinatal depression only among pregnant women with subthreshold depressive symptoms.

**Disclosure of Interest:**

None Declared

